# Supplementary material for: Patterns of Intron Gain and Loss in Fungi
Source: PLoS Biol. 2004 Nov 30;2(12):e422. doi: 10.1371/journal.pbio.0020422 (PMC532390; doi:10.1371/journal.pbio.0020422)
Supplement: Table S1 — Also available at http://genes.mit.edu/NielsenEtAl/. (4.3 MB ZIP). [file pbio.0020422.st001.zip › NielsenEtAl/html/556.html]

AN9103.1.NCU05850.1.MG08262.1.FG02433.1


```
 CLUSTAL W (1.82) Multiple Sequence Alignments - Introns Inserted


Sequence 1: NCU05850.1	547 aa
Sequence 2: MG08262.1	548 aa
Sequence 3: FG02433.1	540 aa
Sequence 4: AN9103.1	561 aa
Alignment Length: 565 aa
Number Identitical Residues: 237 aa
Alignment Score (without introns) 12528


MG08262.1 	MAQEYKLKSVTSLD-LKPGDKQEVEVEGLPDAKVLLVNAAGKIQAIGPKCTHYG~APLVK
NCU05850.1	MAQEYKLKGITSLN-LQPGEKHEVEVEGL-DAKVLLLNAAGKTQAIGPRCTHYG2APLAK
FG02433.1 	MSQEYKLKGLSSLS-LSPGSKQEVEVEGIEGGKVLLVNTGGTTQALGAKCTHYG~APMAK
AN9103.1  	MAQKYKLKDISSLTGINNFDKVEAEVEGVQDGKVLLVKYEDKVHAISPKCTHYG~APLKN
          	*:*:****.::** .:.  .* *.****: ..****::  .. :*:..:***** **: :

MG08262.1 	GVLTTSGRITCPWHGA~CFSATTGDVEDAPALDALPVFKVVERDGAVYVTGDEATIKAGR
NCU05850.1	GVLGHNGKLTCPWHGA~CFNTTTGDIENAPALDALPVFKATERDGAVYITGDAETIKAGH
FG02433.1 	GVLTSDGRITCPWHGA1CFNAKTGDIEDAPALDHLPVFKVAERDGAVYVTGEESAIKSSK
AN9103.1  	GVVSPEGRITCPWHGA1CFNVKSGDIEDAPAPAALNTFELFEENGSVYIRGEESAIKSGQ
          	**:  .*::******* **...:**:*:***   * .*:  *.:*:**: *:  :**:.:

MG08262.1 	RKPKFQCSSVQKGEEGKVVVVGG~GSGCIGLIEELRERGFTGPITVISNEGNLPIDRTKL
NCU05850.1	RKPKIKCKVAGAPLSDRVVVVGG~GSGALGAVEGLRELGFSGPITVVTNEGYLPIDRPKL
FG02433.1 	RQPNVKCAGSSSAQDEHVVVVGG~GSGTLGVVESLREKGWKGGITIVSNEGNYPIDRTKL
AN9103.1  	RISEHKCSSSGPG---GLVIIGG2GSGTLGVILAIRELGYNGAITIITREPSLIIDRTKL
          	* .: :*          :*::** *** :* :  :** *:.* **:::.*    ***.**

MG08262.1 	SKALLTDLAKLQWRDEEFYKSGAVEFVQDEVTGVDFKGKSVETKSKKTISYGKLVLATGG
NCU05850.1	SKTLMTDLSKLQWRDAEWFKTGDIDFVQDEVVGVDFGGKTVKTKSGQQLPYSRLVLATGG
FG02433.1 	SKALLTDLDKLTWRNKDFYESGSIKFVDGHVNNVDFSGRFVTTEGGEKISYTKLVLATGG
AN9103.1  	SKALIPDPEKIQWRSPQWYKDVGIETVSDEVSAVDFSQKIVVTRSGKTFPYTKLVLATGG
          	**:*:.*  *: **. ::::   :. *...*  ***  : * *.. : :.* :*******

MG08262.1 	TPRNLPLEGFKTLDNIFTLRNVHDAKNIVSAIGESKGKKIVVVGSSFIGMEVANATAKDN
NCU05850.1	TPKLLPLQGFQVLGNIFTLRNVHDAKNIKEAIGEKKGKKIVIIGSSFIGMEIAACTSDGN
FG02433.1 	TPRRLPLEGFKNLHNIFTLRTVNDTKKIVDQIG-NKGKKIVVVGSSFIGMECAVATASDN
AN9103.1  	VPRTLPLEGFQLLENVFKLRTVTDVQRILNAIGDGKNKKVVIIGSSFIGMEVGNALSKDN
          	.*: ***:**: * *:*.**.* *.:.* . **. *.**:*::******** . . :..*

MG08262.1 	SVSVVGMEKVPLERVLGDKVGSAAQKGLEKSGVKFYMEAGVEKATAHESDPAKVGAVLLK
NCU05850.1	DVAIIGMEKEPLERVLGERVGAIVRKNIESKGVKFYMSAGVDKAEPSSADPSKVGSVHLK
FG02433.1 	DVTVIGMENVPLERVLGEKVGAGLQKALEGKGVKFYMGASVDKAEPSSSDSSKVGAVCLK
AN9103.1  	EVTIVGQESAPMERVMGTEVGHIFQRNLEKAGVKFKLSAGVAKATPSNEEARKVGAVHLQ
          	.*:::* *. *:***:* .**   :: :*  **** : *.* ** . . :. ***:* *:

MG08262.1 	SGESLPADIVILGVGVAPATEFLRGNAGVQLLQDGSLEVDTAFAVTGVK-DVYALGDIAT
NCU05850.1	DGTKLDADMVILGVGVSPATEYLKENSVVRLEDDGSLKVDESFSVVGLK-DVYAIGDIAS
FG02433.1 	DGTRLEADLVVLGVGVAPATQYLQNNSSITLEKDGSVKVNDDYSVAGLK-DVYAIGDIAT
AN9103.1  	DGTVLPADVVILGVGVRPATDFLQGNPAITLEKDGSIKVDEHFSVPGLNNDVFAIGDIAT
          	.*  * **:*:***** ***::*: *. : * .***::*:  ::* *::.**:*:****:

MG08262.1 	FPYHGPGAG---GKGVRIEHWNVAQKSGREAAKHIVDPAG--------FKAEFFTPIFWS
NCU05850.1	YPYHGPGGD---GKYVRIEHYNVAQNAGRTVANHIVHPN---------LKPEFFTPVFWS
FG02433.1 	FPYHGPGGE---GKPVRIEHWNVAQKAGRIAANHIANPG---------GKTEHFIPIFWS
AN9103.1  	FPYHGPGTDPKKGTYTRIEHWNVAQNAGRSVASSILHMLHNTTSSLQKVKPKVFIPIFWS
          	:******  ...*. .****:****::** .*. * .   .:::: .. *.: * *:***

MG08262.1 	ALSAQLRYCGNTMASGWDDVVIQGSLDEGKWSAFYAKGDEVVAVATMGADPVMVQSAELM
NCU05850.1	ALGAQLRYCGN-SNHGWDDLVLQGEPDQGKWVAYYTKGETVVAMASMGKDPAMAQCAQMM
FG02433.1 	ALGAQLRYCGNTMASGWDDLVLDGSPAENQFIAYYCKGETVVAMASMGRDPAMAQSAELM
AN9103.1  	ALGSQLRYCGN-TIMGWDDLVLKGEPENAKFAAYYCKGETVVAVATMGMDPIMVKCAELM
          	**.:*******    ****:*:.*.  : :: *:* **: ***:*:** ** *.:.*::*

MG08262.1 	RRGKMPKKSELEKGMDVMSLGAPE~----
NCU05850.1	QLNKMPTKSQLKDGLDILSLGPPQ2PL--
FG02433.1 	RVNKMPTKTELQEGISVI------~----
AN9103.1  	RRNNMPSKKEIQDGVDVLSIDVPK~GVRI
          	: .:**.*.:::.*:.::: . ..
```
